# Portal vein flow velocity as a possible fast noninvasive screening tool for esophageal varices in cirrhotic patients

**DOI:** 10.1002/jgh3.12301

**Published:** 2020-01-22

**Authors:** Yara N Elkenawy, Reda A Elarabawy, Layla M Ahmed, Abdallah A Elsawy

**Affiliations:** ^1^ Department of Internal Medicine, Faculty of Medicine Tanta University Tanta Egypt; ^2^ Department of Diagnostic Radiology, Faculty of Medicine Tanta University Tanta Egypt

**Keywords:** cirrhosis, portal vein velocity, screening, varices

## Abstract

**Background and Aim:**

Esophagogastroduodenoscopy (EGD) is the gold standard tool in both screening/diagnosis and management of varices in cirrhotic patients; however, its invasive nature may be uncomfortable to some patients, and in addition, it may be unavailable in some centers that cannot afford it. Therefore, to decrease the economic and physical burden on patients, multiple noninvasive clinical, laboratory, and radiological parameters are evaluated as triage screening predictors of varices before patients' referral to endoscopy. In this respect, we tried to evaluate the validity of portal vein velocity (PVV) as a noninvasive screening tool of esophageal varices (EV).

**Methods:**

One hundred thirty‐five cirrhotic patients were consecutively enrolled in this cross‐sectional study. All patients were evaluated independently and blindly by EGD as the gold standard and then by Doppler ultrasound on portal vein (PV).

**Results:**

Univariate regression showed significant coefficients for PVV, platelet (PLT), albumin, bilirubin, international normalized ratio (INR), portal vein diameter, and ascites; however, multivariable regression showed significant coefficients only for PVV, PLT, and albumin; (*P* = 0.000, 0.000, and 0.006, respectively). Area under the receiver operating characteristic curve (AUROC), sensitivity, specificity, LR+, and LR− values were then calculated and validated using bootstrap analysis. PVV was more accurate than other evaluated parameters (AUROC: 0.927 and *P* = 0.000). The most accurate rule out cutoff value for PVV was ≥19 cm/s with the sensitivity of 97% and LR− of 0.05.

**Conclusion:**

PVV may be useful as a noninvasive triage test for selection of the high‐risk cirrhotic patients who should be referred to and could benefit from EGD. We could highlight using PVV to rule out EV at a cutoff value ≥19 cm/s, reserving EGD only for patients with the PVV value <19 cm/s.

## Graphical abstract

Esophagogastroduodenoscopy (EGD) is the gold standard tool in both screening/diagnosis and management of varices in cirrhotic patients, however, its invasive nature may be uncomfortable to some patients, and in addition, it may be unavailable in some centers with low economic conditions. Solving these obstacles, we tried to evaluate the validity of portal vein velocity as a non‐invasive triage screening tool of esophageal varices before patients' referral to endoscopy so as to decrease the economic and physical burden on patients. We found that portal vein velocity may be useful as a non‐invasive triage test for selection of the high risk cirrhotic patients who should be referred to and could benefit from EGD.

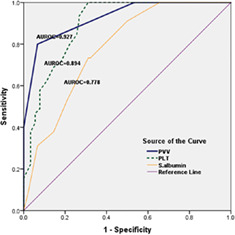

## Introduction

Esophageal varices (EV) are major complications in patients with liver cirrhosis. They are present in decompensated cirrhosis more frequently than in compensated cirrhosis, as it correlates well with the severity of liver disease. Bleeding EV are the most common cause of upper gastrointestinal bleeding in cirrhotic patients; it is a life‐threatening condition with a high mortality rate; approximately 40% of cirrhotic patients with EV may present with variceal bleeding, with a mortality rate of about 20%. In this respect, early screening of EV is highly recommended in high‐risk cirrhotic patients and so appropriate prophylactic treatment should be considered as soon as varices are detected in order to decrease the incidence of EV bleeding.^1^, ^2^, ^3^, ^4^

Esophageal endoscopy is the gold standard for diagnosis of EV and management of the bleeding. It is also used as a screening tool recommended to all cirrhotic patients at the time of initial diagnosis to screen for the presence of EV.^4^ However, esophagogastroduodenoscopy (EGD) was found to have low patient acceptance because of its invasive nature and fear of its complications; in addition, it is not always available in some centers that cannot afford it. Therefore, the use of accurate noninvasive tools like abdominal ultrasound and biochemical predictors is often needed in settings where EGD is not available. These predictors may have significant patient acceptance as EV could be predicted noninvasively in high‐risk cirrhotic patients before invasive diagnostic endoscopy and unnecessary endoscopy could be avoided in low‐risk cirrhotic patients. These predictors have been evaluated by multiple studies without consistent results.^5^, ^6^, ^7^, ^8^

In this study, we tried to evaluate the possible usefulness of portal vein velocity (PVV) during abdominal ultrasound as a fast noninvasive screening modality for EV in cirrhotic patients. We intended to use PVV as triage test before endoscopy to identify high‐risk cirrhotic patients who should be referred to and could benefit from invasive endoscopy.

## Methods

### *Ethical consideration*

The present study was conducted in accordance with the Declaration of Helsinki. The study protocol was approved by the ethics committee of Tanta Faculty of Medicine (32438107118). All patients provided written informed consent. The results of the research were used only for scientific purposes and not for any other aims. The risk of infection during blood sampling or endoscopy was avoided by adhering to complete aseptic and sterilization techniques.

### *Study design*

This study was a cross‐sectional study. This diagnostic study was conducted according to Standards for Reporting of Diagnostic Accuracy Studies (STARD) guidelines.^9^

### *The eligible population*

This study was performed at Internal Medicine and Radiology Departments, Tanta University Hospital (a tertiary hospital), in the period from June 2018 to April 2019 on a total of 243 newly diagnosed chronic hepatitis patients who were consecutively selected from our outpatient clinics.

### *Exclusion and inclusion criteria*

**Figure 1:**
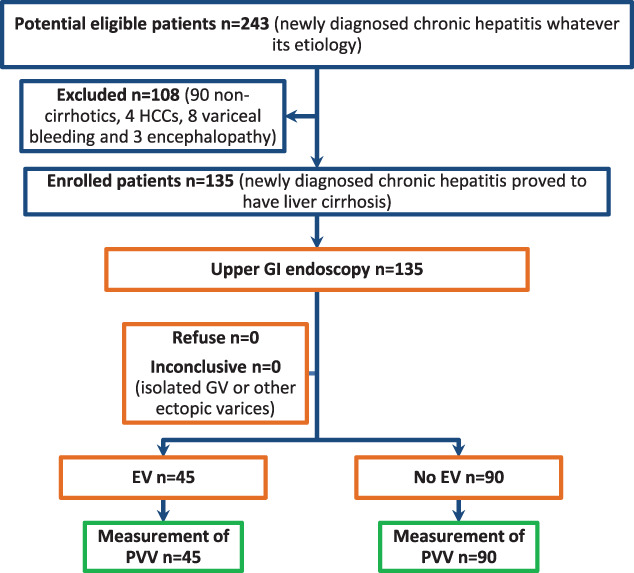
Study flow diagram. EV, esophageal varices; HCC, hepato‐cellular carcinomas; GI, gastrointestinal; GV, gastric varices; PVV, portal vein velocity.

Eighteen patients were excluded because of the presence of cirrhosis complications (4 patients with hepato‐cellular carcinomas [HCCs], 3 patients with portal vein thrombosis, 8 patients with variceal bleeding and 3 patients with encephalopathy), and 90 patients were excluded because the diagnosis of cirrhosis was not confirmed. None of our patients had been receiving beta‐blockers prior to the diagnosis or to EGD examination. The research question of our study targeted cirrhotic patients with EV with or without gastric extension even with isolated gastric varices (GV). Isolated GV or other ectopic varices without EV were not considered as an inclusion criterion in our study because of its much less prevalence than EV and were considered as inconclusive findings; however, none of our included patients had isolated GV or other ectopic varices. Therefore, 135 patients with newly diagnosed liver cirrhosis were included in this study as illustrated in Fig. 1.

### *Assessment of cirrhosis criteria*

All included cirrhotic patients were selected accurately using clinical evaluation, laboratory assessment (serum albumin, serum bilirubin, aspartate amino‐transferase, alanine amino‐transferase, international normalized ratio, and complete blood count), abdominal ultrasound parameters (irregular wavy liver margins, coarse texture, attenuated hepatic veins, prominent caudate lobe, portal vein diameter (PVD), spleen size, and presence of ascites) and liver fibroscan. Liver biopsy was necessary in only two cases to confirm the diagnosis of cirrhosis.

### *Data collection*

Each patient was evaluated according to following 3‐day protocol. In the first day, the evidence of liver cirrhosis was assessed, and the patient was enrolled in the study if he or she met our inclusion and exclusion criteria; in the second day, the patient was referred to our endoscopy unit for esophageal endoscopy for diagnosis and grading of EVs; and in the third day, the patient was referred to abdominal ultrasound unit for Doppler of portal vein. Data collection for each patient was completed within a period of 2 weeks in all patients. All variceal patients did not receive any treatment modality for EV after doing endoscopy and before doing Doppler of portal vein. All operators worked independently and were blinded to the patients' other instrumental, clinical, and laboratory data. All operators were staff physicians with long‐term experience in endoscopy as well as in ultrasound examination of the abdomen.

### *Upper gastrointestinal endoscopy*

A standard endoscopic examination was performed by the same operator, after the patient fasted for at least 6 h and in the morning before lunch time, using an Olympus190 videoscope, Japan. The endoscopic findings were recorded and graded as follows: Grade I, varices were flattened by insufflation; Grade II, varices not flattened by insufflations and separated by areas of normal mucosa; and Grade III, confluent varices not flattened by insufflation.^10^

### *Doppler ultrasound of portal system*

The Doppler of portal system was performed by the same operator to eliminate the interoperator variability; at the same time, it was done in the morning before the lunch time, to eliminate the effects of probable differences in portal pressure in different times of the day. PVV as well as PVD was calculated using the Siemens X 300 device. During the measurement of PVV, the angle between the Doppler beam and the long axis of portal vein should be <60°, the portal vein is imaged longitudinally in the supine position, and the Doppler sample volume was set at its crossing point with the hepatic artery. When the sample point is adjusted to the center of the portal vein, the PVV was recorded in a suspended expiration and was averaged over a few seconds.^11^

### *Statistical analysis of the data*

All collected data were organized, tabulated, and statistically analyzed using the IBM SPSS (IBM corp., NY, USA), version 23, statistics software, IBM corp., NY, USA. For quantitative data, the median and interquantile range (IQR) were calculated as all our collected data were abnormally distributed. Qualitative data were reported as frequency and percentage. Mann–Whitney *U* test was used for two group comparisons for quantitative data. Chi‐Square test was performed to conduct group comparisons for categorical data.

Logistic regression analysis was performed to find the best noninvasive predictor of EV. Candidate predictors considered in this analysis were PVV, PVD, serum albumin, serum bilirubin, INR, platelet count, and ascites. Simple logistic regression analysis was done first for each predictor, and then the best predictor was evaluated using the multivariable logistic regression analysis by entering all predictors simultaneously with a stepwise backward strategy.

Receiver operating characteristic (ROC) curves were calculated for each of the predictors evaluated and each area under the ROC curve (AUROC) was computed. The respective cutoff values were selected according to the aim of the test, that is, to rule in or rule out the presence of EV, choosing respectively the highest positive likelihood ratio (LR) and the lowest negative LR. Sensitivity, specificity, LR+, and LR− with their 95% confidence intervals (CIs) were calculated at each cutoff. The bootstrap method was used for internal validation of our results. *P* values less than 0.05 were considered statistically significant.

## Results

### *Baseline patients characteristics*

The main demographic, clinical, and laboratory criteria for all included patients are illustrated in Table 1. There was no significant difference between variceal and nonvariceal cirrhotic patients as regards age, gender, alanine aminotransferase, aspartate aminotransferase, hemoglobin, and spleen diameter. Chronic hepatitis C (CHC) was the most common etiology of cirrhosis in our patients, with no significant difference between both variceal and nonvariceal patients. The median values of serum albumin, serum bilirubin, INR, and platelets were significantly different between variceal and nonvariceal patients (*P* value = 0.000). Thirty (22.2%) of our enrolled patients had ascites, of which 20 patients had EV and 10 had no EV with a significant difference (*P* value = 0.000). Forty‐one (30%) of our patients were classified as Child's A, 67 (50%) as Child's B, and 27 (20%) as Child's C. Seventeen (38%) of variceal patients had Grade I EV, 15(33%) had Grade II EV, and 13(29%) had Grade III EV. None of our included patients had isolated GV or other ectopic varices.

**Table 1:** Main demographic, clinical, and laboratory criteria for studied population

				Gold standard evidence of EV					*P* value
Variable			Total *n* = 135	Variceal group *n* = 45				Nonvariceal group *n* = 90	
Age (years), median (IQR)			46 (9)	45 (10)				47 (9)	0.228
Male, count (%)			98 (72.6)	32 (71)				66 (73)	0.839
ALT (IU/L), median (IQR)			56 (14)	56 (14)				56 (13)	0.228
AST (IU/L), median (IQR)			62 (10)	59 (11)				63 (10)	0.367
S. albumin (g/dL), median (IQR)			3.2 (0.5)	3.1 (0.4)				3.3 (0.4)	0.000
S.bilirubin (mg/dL), median (IQR)			1.5 (0.4)	1.6 (0.3)				1.5 (0.3)	0.000
INR, median (IQR)			1.7 (0.5)	1.8 (0.6)				1.6 (0.6)	0.000
Hemoglobin (gm/dL), median (IQR)			11 (0.9)	10.7 (0.5)				11 (0.9)	0.213
PLT (10^3^/uL), median (IQR)			156 (60)	110 (52)				165 (29)	0.000
Spleen diameter (cm), median (IQR)			13 (8)	13 (4)				13 (2)	0.534
Ascites	Present	Count (%)	30 (22.2)	20 (44)				10 (11)	0.000
Etiology	CHC	Count (%)	116 (86)	37 (82)				79 (86)	0.418
	CHB	Count (%)	14 (10.4)	5 (11)				9 (10)	
	Unknown	Count (%)	5 (3.6)	3 (7)				2 (4)	
Child‐Pugh Classification	A	Count (%)	41 (30)	5 (11)				36 (40)	0.000
	B	Count (%)	67 (50)	22 (49)				45 (50)	
	C	Count (%)	27 (20)	18 (40)				9 (10)	
PVV (cm/s)		Median (IQR)	18 (6)	Grade I EV *n* = 17	Grade II EV *n* = 15	Grade III EV *n* = 13	All EV *n* = 45	19 (3)	0.000
				17 (5)	12 (6)	9 (5)	12 (8)		
				P1 = 0.004	P2 = 0.000	P3 = 0.091			
PVD (mm)		Median (IQR)	13 (5)	17 (6)				12 (4)	0.000

P1: significance between grade I and II EV, P2: significance between grade I and III EV, P3: significance between grade II and III EV.

ALT, alanine aminotransferase; AST, aspartate aminotransferase; CHB, chronic hepatitis B; CHC, chronic hepatitis C; EV, esophageal varices; INR, international normalized ratio; IQR, interquantile range; PLT, platelets; PVD, portal vein diameter; PVV, portal vein velocity.

### *Screening performance of PVV*

Table 1 showed that the median values of PVV in variceal patients were significantly less than that in nonvariceal patients (*P* value = 0.000); at the same time, PVV decreased significantly in Grades II and III EV in compared with Grade I EV (*P* values = 0.004 and 0.000, respectively), with no significant difference between Grades II and III EV (*P* value = 0.091).

The univariate logistic regression analysis for the PVV in comparable to other evaluated noninvasive predictors, showed significant coefficients for PVV, PLT, albumin, bilirubin, INR, PVD, and ascites (*P* values = 0.000 for all with odds ratio [OR]: 0.544, 0.892, 0.019, 32.327, 12.807, 1.519, and 6.400, respectively). After evaluation of all significant predictors using the multivariate logistic regression analysis, we found that the only significant predictors were PVV, PLT, and albumin (*P* values = 0.000, 0.000, and 0.006, respectively, with adjusted OR: 0.418, 0.862, and 0.006, respectively); of these predictors, PVV has the highest Wald value compared with PLT and albumin (16.5, 15.4, and 7.7, respectively), as illustrated in Table 2.

**Table 2:** Logistic regression analysis for the significant noninvasive predictors of EV

Noninvasive predictor	Univariate analysis				Multivariate analysis					
	B	Wald	*P* value	OR	B	Wald	*P* value	Adjusted OR (95% CI)	Constant	Hosmer–Lemeshow test
PVV (cm/s)	−0.610	31.277	0.000	0.544	−0.872	16.503	0.000	0.418 (0.275–0.637)	50.479	0.989
PLT (10^3^/uL)	−0.114	32.49	0.000	0.892	−0.148	15.431	0.000	0.862 (0.801–0.928)		
S. albumin (gm/dL)	−3.957	22.722	0.000	0.019	−5.159	7.698	0.006	0.006 (0.000–0.220)		
Bilirubin (mg/dL)	3.476	15.964	0.000	32.327						
INR	2.550	12.757	0.000	12.807						
PVD (mm)	0.418	29.811	0.000	1.519						
Ascites (present)	1.856	17.017	0.000	6.400						

CI, confidence interval; EV, esophageal varices; INR, international normalized ratio; OR, odds ratio; PLT, platelets; PVD, portal vein diameter; PVV, portal vein velocity.

**Figure 2:**
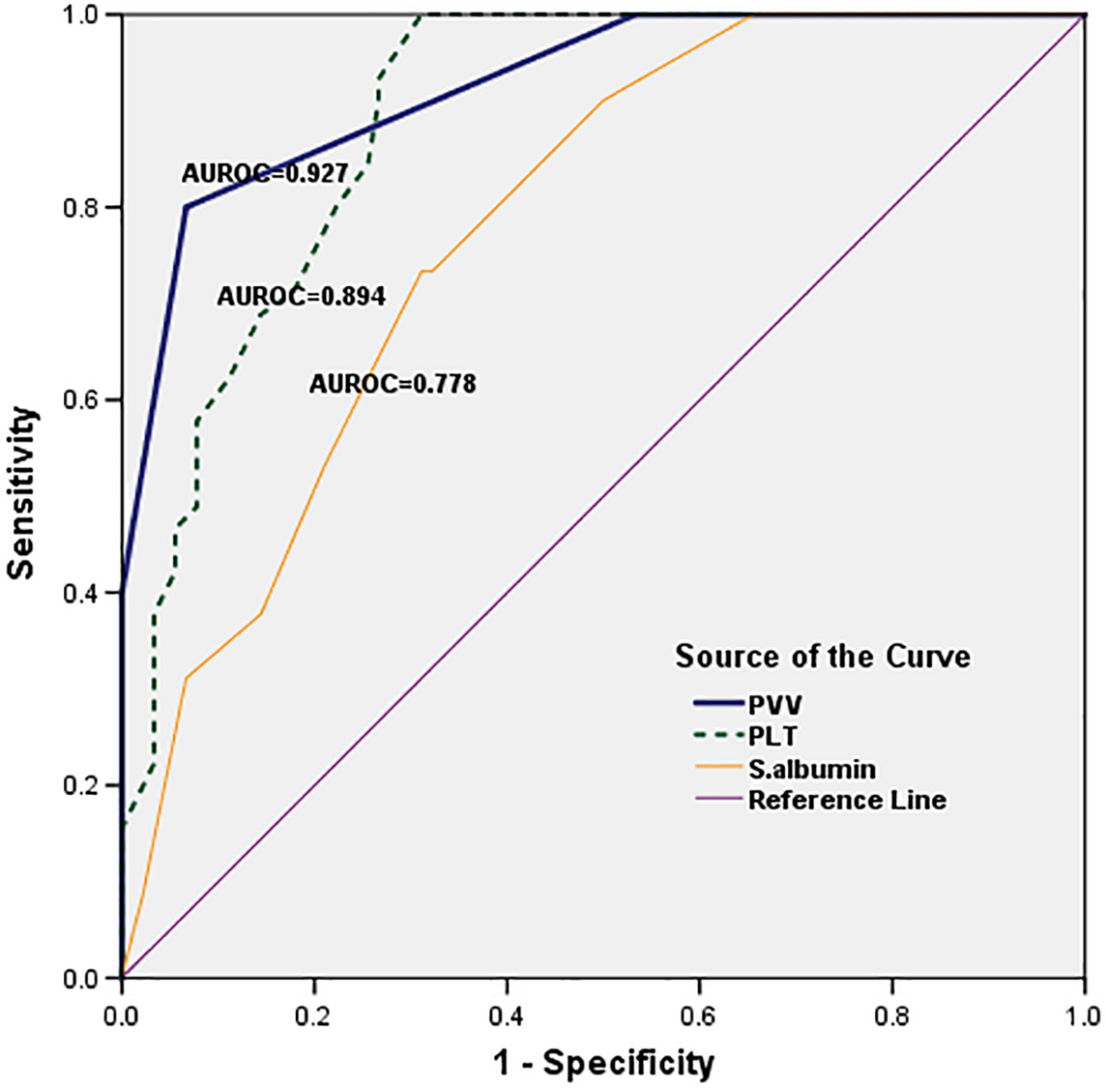
Receiver operating characteristic (ROC) curve for portal vein velocity (PVV), platelets (PLT) and serum albumin. AUROC, area under the ROC curve. (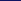) PVV, (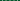), PLT, (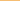), S. albumin, and (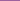), reference line.

Table 3 and Figure 2 showed ROC of the most significant noninvasive predictors of EV (PVV, PLT, and albumin); AUROC of PVV had the largest value (AUROC = 0.927, 0.894, and 0.778 for PVV, PLT, and albumin, respectively). By using ROC curve, two cutoff values were defined for PVV: the first cutoff value (≤7 cm/s) corresponding to the highest LR+ (48) intended to rule in and the other cutoff value (≥19 cm/s) corresponding to the lowest LR− (0.05) to rule out the presence of EV as shown in Table 3.

**Table 3:** Receiver operating characteristics of the most significant noninvasive predictors of EV

Noninvasive predictor	Role	Cutoff	Sensitivity %	Specificity %	LR+	LR−	AUROC		
							Value	(95% CI)	*P* value
PVV (cm/s)	Rule out	≥19	97	40	20	0.05	0.927	0.882–0.971	0.000
	Rule in	≤7	15	99	48	0.9			
PLT (10^3^/uL)	Rule out	≥140	96	27	150	0.05	0.894	0.842–0.945	0.000
	Rule in	≤100	7	99	1515	0.9			
Serum albumin (g/dL)	Rule out	≥3.5	96	55	3.7	0.1	0.778	0.701–854	0.000
	Rule in	≤2.8	20	97	20	0.8			

AUROC, area under the receiver operating characteristic curve; CI, confidence interval; EV, esophageal varices; LR, likelihood ratio; PLT, platelets; PVV, portal vein velocity.

## Discussion

Variceal screening and appropriate prophylactic medical or interventional treatment could decrease the incidence of variceal bleeding and enhance the prognosis of cirrhotic patients' survival. EGD is the gold standard not only in the screening or diagnosis of EV but also in its management; however, it may be limited due to its invasive nature and high cost in some centers, leading to less patients' acceptance; moreover, it may be less available in certain economic conditions. In this respect, many researchers tried to evaluate the validity of many noninvasive radiological, clinical, or laboratory tests to predict EV with debatable results.^12^, ^13^, ^14^, ^15^, ^16^

Doppler evaluation of the portal venous system, including portal and splenic blood velocity and flow, would be of value as a supplementary tool for the evaluation of portal hypertension; however, it is not sensitive enough for its accurate diagnosis because of its conflicting results.^11^

In this study, we evaluated the screening performance of portal vein flow velocity as noninvasive predictor of EV as a triage test to select cirrhotic patients who should be referred to and could benefit from EGD.

Our results highlighted the possibility of using PVV as fast noninvasive screening tool for EV as it is characterized by a high diagnostic accuracy (AUROC = 0.927); moreover, our results highlighted that other examined noninvasive predictors have less prediction accuracy relative to PVV. These observations raise the possibility of using PVV as screening noninvasive test to rule out the presence of EV at a cutoff value ≥19 cm/s with a small LR− of 0.05 with high sensitivity of 97% reserving EGD only for patients with a PVV value <19 cm/s.

Our results are confirmed by comparing with that of Shastri *et al*.^17^ who concluded that PVV could be used as noninvasive triage tests before referral to endoscopy; they found that PVV has the highest sensitivity of 84% at a cutoff level of 16 cm/s in comparison to both PVD and congestion index; however, they used smaller sample size relative to our study 50 *versus* 135 patients that may contribute to the higher value of PVV sensitivity of 97% of our results at a comparable cutoff level of 19 cm/s. Zironi *et al*.^18^ found that PVV of 15 cm/s was the best cutoff value, with a sensitivity and specificity of 88% and 96%, respectively. Moreover, Kayacetin *et al*.^19^ concluded that PVV and portal flow volume decreased with the severity of liver cirrhosis and may predict variceal bleeding risk.

The increased intrahepatic vascular resistance that is reported in liver cirrhosis shares in the etio‐pathogenesis of the decreased PVV that may present in those patients; these data were confirmed by Zironi *et al*. who reported that the mean PVV in cirrhosis was lower than normal subjects (13.0 ± 3.2 *vs* 19.6 ± 2.6 cm/s, respectively).^18^

However, many studies that evaluated the Doppler hemodynamic predictors of EV found that PVV is not a perfect predictor of EV in comparison to other examined parameters in contrast to our results.^20^, ^21^, ^22^, ^23^, ^24^

The near‐to‐normal PVV, which may be reported in some cirrhotic patients with portal hypertension, results from the portosystemic shunts in those patients that may lead to some variability in PVV measurements.^25^, ^26^ Other sources of variabilities in PVV measurement include equipment‐related, intraobserver, and interobserver variability.^27^, ^28^, ^29^ In our study, we tried to decrease the effect of these variabilities through measurement of PVV of all patients by a single highly trained physician at the same time of the day before lunch time, and by using a highly equipped instrument.

The limitation of our study has some aspects; one of these is the difficult to deal variability that may be present due to the underlying portosystemic collaterals; however, we tried to increase the sample size to wash out this effect. Other aspects of study limitations are single center enrollment, which may affect study generalizability, and the single operator, which may lead to intra‐observer variability; however, the highly trained physician could decrease this limitation.

We concluded from our study that PVV may be useful as a noninvasive triage test that may select the high‐risk cirrhotic patients who should be referred to and could benefit from EGD that remains the gold standard for EV diagnosis and management. Our results highlight the possibility of using PVV to rule out the presence of EV at a cutoff value ≥19 cm/s, reserving EGD only for patients with a PVV value <19 cm/s.
